# A Rare Case of Immunotactoid Glomerulopathy Associated with Hodgkin Lymphoma

**DOI:** 10.1155/2021/5527966

**Published:** 2021-05-06

**Authors:** Keiki Nagaharu, Yuka Sugimoto, Keiki Kawakami

**Affiliations:** ^1^Department of Hematology and Oncology, Suzuka General Hospital, Yamanohana 1275-53, Suzuka 513-8630, Mie Prefecture, Japan; ^2^Department of Hematology and Oncology, Mie University Graduate School of Medicine, Edobashi 2-174, Tsu 514-8507, Mie Prefecture, Japan

## Abstract

Immunotactoid glomerulopathy (ITG) is characterized by Congo red-negative microtubular deposits, and it has been reported as a rare paraneoplastic syndrome due to hematologic malignancies, viral infections, or autoimmune diseases. In hematologic malignancies, multiple myeloma and other mature B-cell malignancies are the most common hematologic malignancies, and Hodgkin lymphoma (HL) is extremely rare. A 59-year-old woman was admitted to our hospital because of a pulmonary mass and proteinuria. Computed tomography-guided lung biopsy confirmed the presence of HL stage IIA. Immunofixation of peripheral blood was positive for immunoglobulin G (IgG) kappa. Renal biopsy showed mesangial proliferation with deposits in the subendothelial lesion and no invasion of the HL. These deposits were positive for IgG3, C3, and kappa light chain but negative for C1q and lambda light chain. Electron microscopy showed randomly aligned tubular structures with a diameter of approximately 50 nm. We diagnosed the patient with immunotactoid nephropathy and HL. After systemic chemotherapy, the patient achieved a complete response and loss of proteinuria. On the contrary, her serum monoclonal gammopathy was observed after chemotherapy. The existence of a monoclonal antibody itself might not be a sufficient factor for ITG in some cases, and an additive trigger is necessary for development.

## 1. Introduction

Immunotactoid glomerulopathy (ITG) is an extremely rare condition (approximately less than 1% of renal biopsies [1]) and was first introduced by Schwartz and Lewis [2]. ITG is defined by Congo red-negative microtubular deposits and has been reported as a rare paraneoplastic syndrome due to B-cell lymphoma or multiple myeloma.

Hodgkin lymphoma (HL) is the most common lymphoma in Western countries. Approximately 0.4% of HL cases show glomerular dysfunction, which is mainly diagnosed as minimal change nephrotic syndrome (MCNS) [3]. Serum monoclonal antibodies are normally undetected, but a few cases have been reported as HL with monoclonal gamma globulin [4]. We encountered a case of HL presenting with ITG, and renal biopsy revealed immunotactoid nephropathy (ITG).

## 2. Case Presentation

A 59-year-old woman was admitted to our hospital with chronic cough. Her medical history included glaucoma without medication and a resected benign ovarian cyst. In addition, she had been diagnosed with elevated immunoglobulin G (IgG) levels 3 years prior to admission. Three months before her admission, she noticed a sore throat and cough without sputum. She had been diagnosed with pneumonia because of pulmonary infiltration on chest radiography, and levofloxacin was prescribed in another clinic. However, her symptoms did not resolve, and she was referred to our hospital.

**Figure 1:**
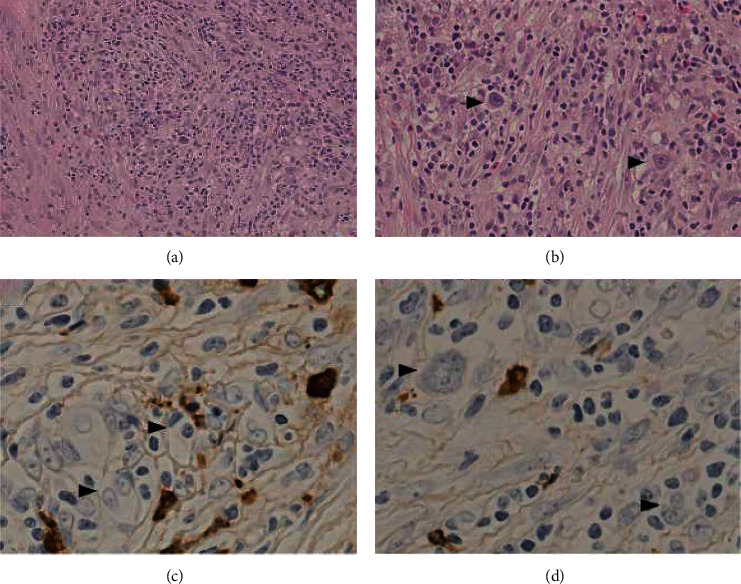
Histopathological evaluation of pulmonary biopsy. (a) HE (×40). (b) PAS (×100). Stained pulmonary biopsy reveals “Hodgkin cells.” Immunofluorescence staining of these Hodgkin cells is negative for kappa (c) and lambda (d).

On admission, chest auscultation revealed attenuated respiratory sounds in the left upper lung. Complete blood counts revealed leukocytosis with predominant neutrophils (12,300/*μ*L (normal, <9,000); neutrophil, 93%). Blood biochemistry revealed hypoalbuminemia (3.3 g/dL), elevated C-reactive protein (10.4 mg/dL), high-soluble interleukin-2 receptor (3,940 IU/dL (normal, <550 IU/dL)), and normal IgG (1,392 mg/dL (normal, <1,700 mg/dL)). However, immunofixation of the serum revealed a low amount of monoclonal IgG kappa. Others including serum electrolytes, blood urea (9.2 mg/dL), creatinine (0.60 mg/dL), IgA (271 mg/dL), IgM (103 mg/dL), C3 (106 mg/dL), and C4 (34 mg/dL) were within normal limits. Serum tests for PR3-ANCA, MPO-ANCA, anti-Ri antibody, anti-Hu antibody, anti-Yo antibody, anti-dsDNA IgG antibody, and anti-dsDNA IgM were all negative. Despite repetitive evaluations, the patient tested negative for cryoglobulin. Antibody tests for hepatitis B virus, hepatitis C virus, and human immunodeficiency virus (HIV) were also negative. Computed tomography (CT) showed a tumor combined with infiltration in the left lower lobe. Pathological findings of CT-guided biopsy of the tumor confirmed stage IIB nodular sclerosis classical HL (Figure 1). Bone marrow specimens revealed a normal population without the proliferation of plasma cells.

**Figure 2:**
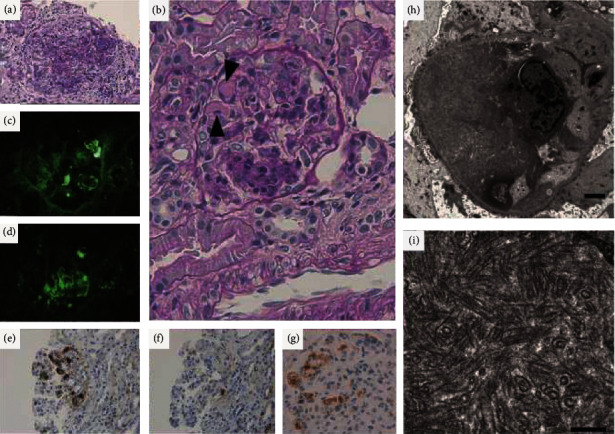
Histopathological evaluation of renal biopsy. (a) PAS (×40). (b) PAS (×100). Stained renal biopsy sections at diagnosis reveal mesangial proliferation and subendothelial deposits (arrowheads). Immunofluorescence staining of these deposits is positive for IgG (c) (×40) and C3 (d) (×40). Immunostaining of the light chain is positive for kappa light chain (e) (×40), negative for lambda light chain (f) (×40), and positive for IgG3 (g) (×40). Using electric microscopy ((h) (×2,500), (i) (×25,000)), these deposits are composed of a tubular structure with a diameter of 50 nm. Black bars in (h) and (i) represent 2.5 *μ*m and 500 nm, respectively.

Despite normal eGFR, her urine was positive for protein, and the estimated daily urine protein amount was 1.36 g/g. Urinary protein was mainly albumin, and urinary monoclonal gamma globulin was absent. Renal biopsy revealed mesangial proliferation and a thickened glomerular basal membrane. PAS-positive and Congo red-negative deposits were observed in the glomerular capillary walls (Figures 2(a) and 2(b)). Immunofluorescence staining was positive for IgG and C3 in the deposits (Figures 2(c) and 2(d)). Immunohistochemistry showed positivity for kappa light chain (Figure 2(e)) and IgG3 (Figure 2(g)) and negative for lambda light chain (Figure 2(f)), IgG1, IgG2, IgG4, and C1q (data not shown). Electron microscopy indicated randomly aligned tubular structures located in the subendothelial and mesangial areas of the glomeruli (Figures 2(h) and 2(i)). The diameter of these tubular structures was approximately 50 nm. We finally diagnosed her renal findings as ITG.

She was treated with six courses of ABVD (Adriamycin 25 mg/m^2^, bleomycin 10 IU/m^2^, vinblastine 6 mg/m^2^, and dacarbazine 375 mg/m^2^ on days 1 and 15, every 4 weeks) without significant adverse events, and the pulmonary lesion disappeared quickly. Urinary protein was negative after two courses of ABVD. However, her serum monoclonal IgG kappa light chain was detected.

## 3. Discussion

The association between HL and nephrotic syndrome is rare (approximately 0.4% in adults) [3]. Among patients with HL with nephrotic syndrome, MCNS is observed as a paraneoplastic manifestation [5]. However, renal pathological findings in our case confirmed membranoproliferative glomerulonephritis with nonamyloid deposits, which was finally diagnosed as ITG. The distinction between ITG, fibrillary glomerulonephritis, and type I cryoglobulin nephropathy has been controversial because of their similar clinical and pathological features. In fact, some ITG cases are not distinguished from the others. Our case is compatible with ITG in view of the restricted light chain and tubular diameter of the deposit. On the contrary, random alignment of tubular deposits and small-vessel thrombosis in renal biopsy were characteristics of cryoglobulin nephropathy. However, there were no cryoglobulin-related peripheral symptoms, and serum cryoglobulin was repetitively negative. We diagnosed ITG rather than cryoglobulin nephropathy.

ITG is a rare disease that accounts for only 0.06% of all kidney biopsies [6]. To date, at least 39 patients have been published as case reports (Table 1). The peak of occurrence is at 60 years of age, and deposits are IgG dominant (approximately 70%), which is compatible with a previous report [7]. Hematologic malignancies, including multiple myeloma, lymphoplasmacytic lymphoma, and diffuse large B-cell lymphoma, have been reported. These cases were also positive for serum monoclonal immunoglobulins. However, T-cell lymphoma [8] and mycosis fungoides [9] have been reported as ITG cases. Monoclonal gammopathy was negative in these cases. In addition, several types of underlying diseases other than hematologic malignancies include tuberculosis [10], HIV infection [11–13], and hepatitis C virus infection [14, 15]. To the best of our knowledge, this is the first reported case of HL with ITG.

In our case, we suspected that ITG nephropathy was induced as a paraneoplastic syndrome. Hudnall et al. [4] described a case of HL with heavy chain monoclonal gammopathy, and they suggested three possible pathogeneses of monoclonal gammopathy in HL: (1) two distinct neoplastic processes, (2) produced by Hodgkin cells, or (3) a single neoplastic process leading to two subclones (one producing HL and the other producing gammopathy). As for the second possibility, we performed additional immunohistochemical staining of HL cells in pulmonary lesions, which was negative for light chain expression (Figures 1(c) and 1(d)). As for the first and third Hudnall et al.'s hypotheses, our patient's medical history included elevated IgG, but the etiology was unknown. As HL itself generally does not accompany monoclonal gammopathy, our case might have underlying monoclonal gammopathy before the onset of HL. The exact mechanism is uncertain, but HL may produce some trigger substances for the development of ITG. In conclusion, we encountered a rare case of HL presenting with ITG. We could not reveal the exact mechanism, and further development of the pathological elucidation of ITG is desired.

## Figures and Tables

**Table 1:** Literature review of previous ITG case reports (available 39 cases).

Age and sex		Hematologic underlying disease	No.
Age	Median 60 (6–79)	Multiple myeloma	5
Sex	Male, 56%; female, 44%	Lymphoplasmacytic leukemia	4
		Chronic lymphocytic leukemia	3

Component of deposits		Mycosis fungoides	1
IgG	69%	POEMS	1
IgM	30%	AITL	1
IgA	7%	HES	1

The literature search was performed using PubMed with the following medical keywords: {immunotactoid nephropathy} or {immunotactoid glomerulopathy}. Among 136 articles, we adapted the reports with at least one of the following data: age and sex, deposit immunoglobulin, and hematologic underlying diseases.

## Data Availability

No data were used to support this study.
